# Validity of the Psychosocial Impact of Dental Aesthetics Questionnaire for use on Brazilian adolescents

**DOI:** 10.1590/2177-6709.21.3.067-072.oar

**Published:** 2016

**Authors:** Paula Mendes Santos, Alcides Ricardo Gonçalves, Tatiane Marega

**Affiliations:** 1 MSc, Faculdade São Leopoldo Mandic, Dentistry Specialization for Special Needs Patients, Campinas, São Paulo, Brazil.; 2 Professor, Faculdade São Leopoldo Mandic, Dentistry Specialization for Special Needs Patients, Campinas, São Paulo, Brazil.; 3 PhD Professor, Faculdade São Leopoldo Mandic, Dentistry Specialization for Special Needs Patients, Campinas, São Paulo, Brazil.

**Keywords:** Validation study, Dental aesthetics, Malocclusion, Quality of life, Dentistry.

## Abstract

**Introduction:**

The Psychosocial Impact of Dental Aesthetics Questionnaire (PIDAQ) is a multi-item psychometric instrument used to assess patients' perspective of the impact specifically related to Orthodontics. The cross-culturally adapted Brazilian version of the PIDAQ has demonstrated good reliability, validity and acceptability.

**Objective:**

The aim of the present study was to test the validity and reliability of the Brazilian version of the PIDAQ for use among adolescents aged between 11 and 14 years old.

**Methods:**

Having established the possibility of maintaining the operational characteristics of the Brazilian version of PIDAQ for the target age group, 194 individuals in the city of Belo Horizonte, Brazil, completed the questionnaire. The subjects were examined for the presence/absence of malocclusion based on the criteria of the Dental Aesthetic Index (DAI) to test discriminant validity. Internal consistency was measured by means of Cronbach's alpha coefficient which ranged from 0.59 to 0.86 for the subscales. Test-retest reliability was assessed by means of intraclass correlation coefficient which ranged from 0.54 to 0.89 for aesthetic concern and psychological impact.

**Results:**

Discriminant validity revealed that subjects without malocclusion had different PIDAQ scores in comparison to those with malocclusion.

**Conclusion:**

These findings suggest that the Brazilian version of PIDAQ for adolescents has satisfactory psychometric properties and is applicable to this age group in Brazil.

## RESUMO

**Introdução:**

o *Psychosocial Impact of Dental Aesthetics Questionnaire* (PIDAQ) é um instrumento psicométrico utilizado para avaliar a qualidade de vida relacionada à saúde bucal (QVSB), especificamente no que diz respeito à má oclusão do paciente. A versão brasileira adaptada culturalmente do PIDAQ demonstrou boa confiabilidade, validade e aceitabilidade.

**Objetivo:**

o objetivo do presente estudo foi verificar a validade e confiabilidade da versão brasileira do PIDAQ para uso em adolescentes brasileiros na faixa etária de 11 a 14 anos.

**Métodos:**

tendo-se estabelecido a possibilidade de manter a versão brasileira do PIDAQ que havia sido validada, esse questionário foi aplicado em 194 indivíduos, da faixa etária-alvo, residentes na cidade de Belo Horizonte, Brasil. Para testar a validade discriminante, os adolescentes foram examinados quanto à presença ou ausência de má oclusão, com base nos critérios do *Dental Aesthetic Index* (DAI). A confiabilidade foi medida pela consistência interna do instrumento (alfa de Cronbach = 0,59-0,86) e pelo teste-reteste, por meio do coeficiente de correlação intraclasse (ICC), que apresentou valores superiores a 0,8 para os domínios autoconfiança, impacto social e impacto psicológico, o que indica uma concordância satisfatória entre as avaliações.

**Resultados:**

a validade discriminante revelou uma diferença estatisticamente significativa entre os escores médios para os domínios de autoconfiança dentária e impacto psicológico, entre os grupos com e sem má oclusão.

**Conclusão:**

esses resultados sugerem que a versão brasileira do PIDAQ para adolescentes tem propriedades psicométricas satisfatórias e é aplicável a esse grupo etário no Brasil.

## INTRODUCTION

Oral health-related quality of life (OHRQoL) measures are important tools to evaluate the impact of oral problems on one's daily life, since affected individuals seem to be the best people for judging their own quality of life.^1^ ^,^ ^2^Judgment regarding facial attraction is greatly influenced by the appearance of the smile. Therefore, malocclusion can exert a negative effect on psychological well-being and social interactions.^3^

As malocclusion is not a disease, but a misalignment of the teeth, the limit between acceptable and unacceptable occlusion, as well as decisions regarding when orthodontic treatment is desirable, are influenced by one's self-rated dental appearance. Differences in self-perceived dentofacial aesthetics are due to subjective considerations, self-esteem, sex, age group and socioeconomic background.^4^

The age of 11 to 14 years old marks the period of early to mid adolescence, which is equivalent to the onset of puberty. In this phase, adolescents assign significant importance to their physical appearance and perceive the negative aesthetic effects of malocclusion.^4^ ^,^ ^5^ ^,^ ^6^Thus, it is important to understand the implications of the biopsychosocial aspects of malocclusion with regard to adolescents' quality of life, especially because many of these individuals do not have access to orthodontic treatment. In an investigation involving 403 Brazilian adolescents from public and private schools, 69% of parents reported that their children could not undergo orthodontic treatment due to the financial costs involved.^7^ Thus, OHRQoL assessment tools can assist planning of resources to access orthodontic treatment through a better assessment of treatment priorities.

The Psychosocial Impact of Dental Aesthetics Questionnaire (PIDAQ) is a multi-item psychometric tool used to assess patients' perspective of impact specifically related to Orthodontics.^8^ The cross-culturally adapted Brazilian version of PIDAQ has demonstrated good reliability, validity and acceptability.^9^ Although the instrument was developed to be applied to young adults, its applicability to adolescents has been suggested.^8^ Therefore, the aim of the present study was to test the validity and reliability of the Brazilian version of PIDAQ for use on adolescents aged between 11 and 14 years old.

## MATERIAL AND METHODS

### Description of PIDAQ

The PIDAQ is an orthodontic-specific OHRQoL measure used to assess the psychosocial impact of dental aesthetics on young adults. This self-rating instrument is composed of 23 items distributed among four subscales: aesthetic concern (three items), psychological impact (six items), social impact (eight items) and dental self-confidence (six items). Each item is scored on a five-point scale with the following response options: "not at all" = 0; "a little" = 1; "somewhat" = 2; "strongly" = 3; and "very strongly" = 4. A score of 0 indicates no impact of dental aesthetics on OHRQoL while a score of 4 indicates maximum impact.^8^

### Classification of malocclusion

Malocclusion was classified by means of the Dental Aesthetic Index (DAI) criteria, which provide assessment based on socially defined occlusal standards of dental aesthetics and is recommended by the World Health Organization.^10^ DAI has four possible outcomes regarding orthodontic treatment needs: a score ≤ 25 indicates zero or minor malocclusion for which no treatment is needed; a score ranging from 26 to 30 indicates definite malocclusion for which treatment is elective; from 31 to 35, it indicates severe malocclusion for which treatment is highly desirable; while a score greater than 36 indicates handicapping malocclusion for which treatment is mandatory.^11^

### Pilot study

To determine the possibility of maintaining the operational characteristics of the Brazilian version of PIDAQ,^9^ such as the format of the instrument, instructions, measurement methods, number of questions, response options and self-applicability,^8^ a pilot study was conducted with 20 subjects (11 boys and 9 girls, all aged 12 years old), enrolled at a public school in the city of Belo Horizonte, Minas Gerais, Brazil. Results demonstrated that there was no need to change the proposed methods, suggesting operational validity. Subjects in the pilot study were not included in the main sample.

### Assessment of validity and reliability of the Brazilian version of PIDAQ for use on adolescents (PIDAQ-A)

The validity and reliability assessments of PIDAQ-A were carried out in the city of Belo Horizonte, Minas Gerais, Brazil. To assess the psychometric properties of the questionnaire, a convenience sample of 194 adolescents aged between 11 and 14 years old was recruited from a public school.

The exclusion criteria were: intellectual and/or physical inability to answer the questionnaire, previous orthodontic treatment, presence of carious lesions with cavities, moderate to severe fluorosis (dark areas) or pigmented spots in the anterior region and missing or fractured teeth.

The 194 adolescents aged between 11 and 14 years old completed the PIDAQ-A at school and were examined for malocclusion based on DAI. A single examiner who had been previously trained and calibrated for the use of the index performed the examinations (weighted Kappa = 0.58 to 1.00 and intraclass correlation coefficient (ICC) = 0.64 to 1.00 for malocclusion). For statistical purposes, the subjects were separated into two groups: absent (DAI ≤ 25) or present (DAI > 25) malocclusion. 

This study received approval from the Human Research Ethics Committee of Universidade Federal de Minas Gerais (UFMG), Brazil (ETIC 109/08). Only adolescents and parents/guardians who agreed to participate by signing a statement of informed consent were included in the study. 

### Statistical analysis 

Statistical Package for Social Sciences (version 20.0, SPSS Inc., Chicago, Illinois, USA) was used for data analysis. Simple descriptive statistics were generated to characterize the sample (mean, median, standard deviation, analysis of total and individual PIDAQ subscale scores to generate total and subscale scores for each participant). Kolmogorov-Smirnov test revealed that the data exhibited non-normal distribution. Therefore, nonparametric Mann-Whitney test was used to assess differences in the mean scores between groups. 

Internal consistency of PIDAQ subscales for use on Brazilian adolescents was tested by means of Cronbach's alpha coefficient.^12^ All individuals in the sample responded to the questionnaire a second time after a two-week interval. Test-retest reliability was assessed by calculating intraclass correlation coefficient (ICC) with a two-way random effect model for the PIDAQ score. Discriminant validity was tested by comparing the two categorized groups based on DAI (malocclusion absent or present) and each subscale of PIDAQ. The level of significance was set at 5%.

## RESULTS

A total of 194 adolescents aged between 11 and 14 years old (mean age 13 years ± 1.07 years) comprised the sample. Sex was evenly distributed, with 104 females (53.6%) and 90 males (46.4%). A total of 120 individuals (61.9%) were diagnosed with normal or minor malocclusion, 37 (19%) exhibited definite malocclusion, 19 (9.8%) had severe malocclusion and 18 (9.3%) had very severe or handicapping malocclusion. When the DAI variable was dichotomized, 61.9% had no malocclusion (DAI ≤ 25) and 38.1% exhibited definite, severe or handicapping malocclusion. The minimum and maximum DAI scores were 13 and 53, respectively. Regarding PIDAQ subscales, 4% reported aesthetic concerns, 7.9% reported psychological impact, 9.5% reported social impact and 10.5% reported some impact on dental self-confidence.

### Reliability

Cronbach's alpha coefficient for the subscales ranged from 0.59 for aesthetic concern to 0.86 for dental self-confidence, indicating acceptable to excellent internal consistency. The ICC for test-retest reliability (determined by the reapplication of the questionnaire to all 194 adolescents after a two-week period) ranged from 0.54 to 0.89 for aesthetic concern and psychological impact, respectively (Table 1).

**Table 1:** Reliability statistics for subscales (n = 194).

Variable	Number of items	Cronbach's alpha	Intraclass correlation coefficient (95% confidence interval)*
Aesthetic concern	3	0.59	0.54 (0.39; 0.65)
Psychological impact	6	0.79	0.89 (0.85; 0.92)
Social impact	8	0.77	0.84 (0.79; 0.88)
Dental self-confidence	6	0.86	0.82 (0.74; 0.85)

### Discriminant validity

Based on the dichotomization of DAI results, statistically significant differences between the two groups were found in the median scores for dental self-confidence, psychological impact and social impact (Table 2).

**Table 2:** Discriminant validity: subscale scores for adolescents according to dental aesthetic index categorization.

Psychosocial Impact of Dental Aesthetics Questionnaire	Malocclusion category				
	Normal or minor (n = 120)		Malocclusion (n = 74)		
	Mean ± SD	Median	Mean ± SD	Median	p-value*
		(interquartile range)		(interquartile range)	
Aesthetic concern	3.96 ± 4.30	4.0 (5.0)	4.27 ± 3.21	4.0 (6.0)	0.197
Psychological impact	7.04 ± 5.31	6.0 (7.0)	9.30 ± 5.79	9.0 (9.25)	0.006
Social impact	8.85 ± 6.53	8.0 (8.0)	10.59 ± 6.64	10.0 (8.5)	0.05
Dental self-confidence	11.34 ± 6.29	12.0 (9.0)	9.15 ± 6.26	8.0 (10.0)	0.015

* Mann-Whitney test.

*Two-way random effects model: *p* < 0.001 for all values.

## DISCUSSION 

A number of factors influence growth and development of the jaws and can result in malocclusion, such as genetics, congenital malformation, systemic causes and environmental factors.^13^ There has been increasing interest in the impact of malocclusion on psychosocial well-being, since aesthetic appearance plays an important role in social interactions.^14^ However, the normative need observed by dentists does not always coincide with patient's perceptions. It is therefore important to evaluate an individual's subjective needs, which can be achieved through the administration of questionnaires. As multifaceted disorders have different risk factors working together, it is important to consider potential correlations with confounding variables.^15^ In comparison to other OHRQoL instruments classified as generic measures, PIDAQ is a valuable tool for assessing the impact of malocclusion, since this questionnaire is condition-specific and is able to discriminate more strongly between individuals with different degrees of dental aesthetics.^1^ ^,^ ^8^ PIDAQ has been translated, cross-culturally adapted and validated for use on Brazilian young adults aged between 18 and 30 years old.^9^ However, its applicability to adolescents needs to be tested, as bodily changes occur during adolescence, resulting in developmental changes in one's body concept, as suggested by the authors of the original questionnaire.^8^

Regarding the internal consistency of PIDAQ-A, Cronbach's alpha coefficient ranged from 0.59 for aesthetic concern to 0.86 for dental self-confidence. These values allow comparison between groups for all subscales as well as on the individual level for dental self-confidence. Cronbach's alpha coefficient ranging from 0.5 to 0.7 is generally considered satisfactory for comparisons between groups, and coefficients higher than 0.85 are sufficiently reliable for comparisons on the individual level.^16^ The original instrument presented a lower coefficient for social impact (α = 0.86) and higher coefficient for dental self-confidence (α = 0.91). Other versions of PIDAQ presented a lower coefficient for aesthetic concern (α = 0.75)^9^ and a higher coefficient for social impact (α = 0.95).^17^ Similar results were obtained by the Nepalese, Spanish and Chinese versions.^18^ ^,^ ^19^ ^,^ ^20^

The ICC ranged from 0.54 for aesthetic concern to 0.89 for psychological impact. These coefficients demonstrate adequate to excellent reliability.^21^ The results of test-retest reliability demonstrated lower stability for the aesthetic concern subscale. As this subscale addresses dissatisfaction with one's own dental appearance when looking in a mirror or at photographic and video images, these differences can probably be attributed to the ambivalence and instability that characterise adolescence.^22^

The discriminant validity of the questionnaire (evaluated by correlations between PIDAQ subscales and treatment needs determined by DAI) revealed significant correlations with dental self-confidence, psychological impact and social impact. The psychological impact subscale had the strongest correlation, possibly because comparison processes between individuals play a significant role in psychological well-being, as the aim of this subscale is to measure feelings of unhappiness and inferiority in comparison to others considered to have better dental aesthetics.^17^ ^,^ ^23^

The esthetic concern subscale was unable to detect differences between subjects with and without orthodontic treatment need, probably because 61.9% of the individuals did not require orthodontic treatment. The Brazilian and Spanish versions of PIDAQ also found no correlation between this subscale and DAI.^9^ ^,^ ^19^

Self-consciousness may explain differences in self-evaluations, as some individuals are bothered by minor irregularities, while others with severe malocclusions may be indifferent or even satisfied with their dental aesthetics. These particularities could explain why some individuals are dissatisfied with their dental aesthetics both before and after treatment, while others are indifferent or satisfied at either time.^24^ ^,^ ^25^ Since no statistically significant correlation was found between DAI and the aesthetic concern subscale, and the social impact subscale was at the threshold of significance, it is plausible that the present sample had a low degree of self-consciousness. Such individuals are less self-critical and less prone to self-dissatisfaction.^25^ Another possible explanation for this finding is that the study population was composed of adolescents who may not have mature, stable, objective opinions regarding their aesthetic appearance. Such individuals may be satisfied at times and have a different opinion at other times without the occurrence of any clinical change.^26^

Many adolescents seek orthodontic treatment in early adolescence^27^ and the literature shows that normative needs do not always correspond to patient's perceptions.^28^ This was confirmed by Klages et al^8^ who found greater differences in PIDAQ scores when the results were based on self-assessment in comparison to the rating of the interviewer. It is therefore important to set priorities for health care based on knowledge of patients' psychosocial well-being, as Brazil is a country with considerable social disparities and public healthcare services around the country only offer limited orthodontic treatment.^5^

## CONCLUSION 

The Brazilian version of PIDAQ for adolescents demonstrated good reliability, validity and psychometric properties, which lend support to the use of this questionnaire as a valuable measure for assessing the psychosocial impact of dental aesthetics related to malocclusion among Brazilian adolescents.
